# Evaluating changes in growth and pigmentation of *Cladosporium cladosporioides* and *Paecilomyces variotii* in response to gamma and ultraviolet irradiation

**DOI:** 10.1038/s41598-022-16063-z

**Published:** 2022-07-15

**Authors:** Jesse Bland, Lisa Astuto Gribble, Michael C. Hamel, Jeremy B. Wright, Garrett Moormann, Marlene Bachand, Ginger Wright, George D. Bachand

**Affiliations:** 1 Center for Global Security and Cooperation, Sandia National Laboratories, Albuquerque, NM 87185 USA; 2 Center for Monitoring Systems and Technology, Sandia National Laboratories, Albuquerque, NM 87185 USA; 3 Center for Integrated Nanotechnologies, Sandia National Laboratories, Albuquerque, NM 87185 USA

**Keywords:** Environmental microbiology, Fungi, Microbial ecology

## Abstract

Melanin-containing fungi (black molds) have the capacity to thrive under extreme environmental conditions such as the elevated radiation levels inside the former Chernobyl reactors. These fungi have been hypothesized to grow toward and use gamma radiation as an energy source, but the literature does not clearly address which energies of the electromagnetic spectrum, if any, positively affect fungal growth. The goal of this work was to characterize the response of non-melanized and melanized fungi to two distinct electromagnetic wavelengths, i.e., ultraviolet (UV) and gamma ray, keeping absorption and other potentially confounding variables constant. Exposure to UV or gamma radiation induced significant changes in fungi pigmentation, but not growth rate of *Cladosporium cladosporioides* and *Paecilomyces variotii*. Specifically, increased pigmentation of both fungi was observed in samples exposed to UV, while decreased pigmentation was observed for gamma-irradiated samples. These results provide new insights into the role of electromagnetic energies on growth of fungi and provide an impetus to examine additional energies and types of radiation to develop a fundamental understanding of this phenomenon.

## Introduction

Species of fungi have the ability to thrive in harsh environments that include high levels of heat, cold, salinity, desiccation, acidity, pressure, and radiation^1,2^. One of the most dramatic examples of extremophile fungal survival in radioactive environments is the identification of approximately 2000 strains (arising from 200 species) of fungi in the fourth reactor block of the Chernobyl Nuclear Power Plant (ChNPP)^3^. The catastrophic Chernobyl explosion on 26 April 1986 released approximately 108 Curies of radionuclides into the environment, rendering the area uninhabitable to this day^3^. The discovery of fungi in such highly-radioactive environments is not new; earlier studies have identified the existence of fungi in other highly radioactive areas including nuclear testing sites, reactor cooling pools, high-altitude areas of the Arctic and Antarctica, as well as deep space, including the International Space Station, where nutrients are scarce^4–12^.

Many radiotolerant fungi share a distinctive trait, the production of melanin. Melanin, a high molecular weight blackish pigment constitutively synthesized by the fungi, is concentrated in the fungal cell wall and assembled into multiple concentric layers approximately 100 nm thick consisting of closely packed smaller particles^13,14^. Large amounts of melanin in the fungal cell walls confer upon the fungus an ability to tolerate extreme environments by interacting with a wide range of electromagnetic radiation frequencies. Fungal melanin plays a role in multiple biological functions, e.g., enhancing strength of appressoria during host invasion^15^, as well as photoprotection, energy harvesting, and thermoregulation by readily absorbing and transducing such electromagnetic radiation^4^. Moreover, studies suggest the process of radiosynthesis^16^ may be analogous to photosynthesis, where melanin serves as the analog to chlorophyll. Here, melanin has been hypothesized to harvest and convert energy from ionizing radiation into chemical energy for use by fungi, similar to how chlorophyll uses visible light to harness energy^17^. At this time, however, the exact mechanisms of this process are not fully understood.

Melanized fungi have been found to account for the majority of isolates collected from the surrounding 10-km ChNPP zone, which included damaged buildings, soil, and graphite from the reactor element^18^. The most frequently isolated melanized fungi from the site were *Acremonium strictum* W. Gams, *Aspergillus niger* van Tieghem*, Aspergillus versicolor* (Vuillemin) Tiraboschi, *Alternaria alternata (*Fr.) Keissl*.*, *Aureobasidium pullulans* (de Bary) G. Arnaud*, Cladosporium sphaerospermum* Penz*.*, *Cladosporium cladosporioides* (Fres.) de Vries, *Cladosporium herbarum* (Pers.) Link*, Penicillium hirsutum* Dierckx, and *Penicillium aurantiogriseum* Dierckx. It was observed that the greatest number of melanized fungi were isolated from the remains of the power plant rooms, where the greatest radioactive contamination occurred. Moreover, predominance of some species over others correlated to their ability to survive in higher or lower radiation levels^19^.

Melanized fungi are not only resilient against high-radiation environments, but studies suggest they may be attracted to it. The phenomenon of positive fungal “radiotropism,” or the directed and enhanced growth of fungal hyphae towards sources of ionizing radiation, was reported by Zhdanova et al.^3^ in the mycelium of various melanized fungal strains isolated from a 10-km zone surrounding the ChNPP following the 1986 accident^20^. Of those tested, eighteen of 27 (66%) fungal hyphae isolates exposed to beta and gamma emissions demonstrated positive stimulation of growth towards the radiation source. Of the fungal species isolated from the ChNPP zone, 86% showed positive directional growth^20^. The isolated fungi that showed a directional growth toward the radiation source included *Cladosporium cladosporioides*, *Cladosporium sphaerospermum*, *Purpureocillium lilacinum* (*Paecilomyces lilacinus*) (Thom) Luangsa-ard, Houbraken, Hywel-Jones & Samson), *Penicillium hirsutum* Dierckx, *Penicillium lanosum* Westling, *Penicillium roseopurpureum* Dierckx, and *Penicillium westlingii* Zalessky. However, a similar study by Karpenko et al.^21^ showed only 19% of 47 species of melanized fungi isolated from the same ChNPP zone demonstrated positive radiotropism when exposed to a Cs-137 gamma ionizing radiation source, as well as white light. Moreover, the authors found that both melanized and non-melanized fungi demonstrated positive radiotropism, complicating the key conclusions regarding the relationship between melanin and radiotropism^21^.

The goal of the current work was to characterize the response of non-melanized, non-radiotrophic (*Paecilomyces variotii* Bainier) and melanized, radiotrophic (*C. cladosporioides*) fungi to two different electromagnetic wavelengths, keeping absorption and other potentially confounding variables constant. While the isolates used in our experiments were collected from facilities at Sandia National Laboratories, both of the fungal species were reported to inhabit the ChNPP^22^. To date, the electromagnetic energies used in experiments examining radiotropism have varied widely, and the differences in radiotrophic response to these energies remains poorly understood. As an example, Shuryak et al.^23^ found that fungal growth depended on the energy of X-rays while Dadachova et al.^17^ found that electron transfer properties of melanin in the fungi increased by the same rate regardless of the photon energy. Our study examined the growth rate and pigmentation of *C. cladosporioides* and *P. variotii* in the presence of (i) ultraviolet (UV) light in the 300–350 nm range and (ii) gamma rays produced by a Cs-137 source, which are predominantly 662 keV, while maintaining an equivalent amount of energy deposition on the fungal target from each source. We hypothesized that these two species would display different responses in growth and pigmentation following gamma or UV irradiation based on their intrinsic differences in radiotrophic behavior and melanin production. While changes in growth rate were not observed, significant differences in the pigmentation of both fungi were observed with an increased and decreased level of pigmentation in response to UV and gamma irradiation, respectively. Despite the lack of a positive response to radiation, our findings provide additional insights on the interaction and response of filamentous fungi to electromagnetic radiation. Future studies examining the response of fungi to a defined range of energies, as well as to different types of radiation (e.g., neutron, beta) will enable a deeper fundamental understanding of the properties of electromagnetic radiation that are critical to radiotolerance, radiotropism and radiosynthesis in fungi.

## Results and discussion

### Gamma source and dose modeling

The general literature contains conflicting results on whether the energies of photons interacting with fungi affects the radiotrophic response. As such, we sought to control critical variables while irradiating the fungi with ionizing radiation from a sealed Cs-137 source and a UV source. The Cs-137 source emitted a photon at 662 keV along with other lower energy photons near 30 keV (Table S1).

**Figure 1:**
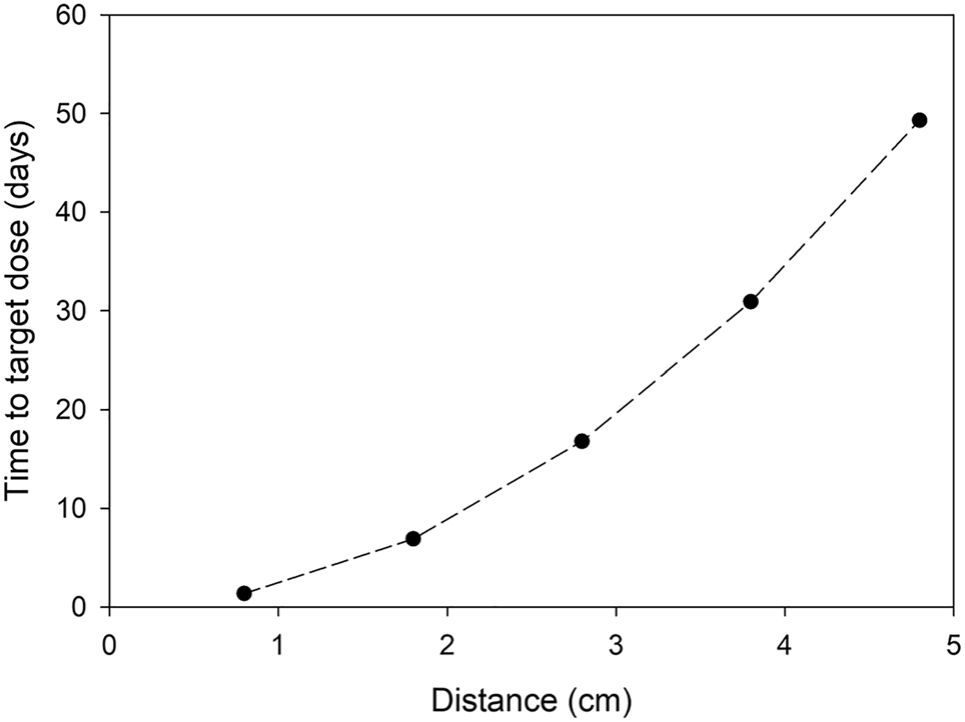
Time required on target to achieve an exposure of 50 rad determined in MicroShield and based on an activity of ~ 350 µCi for Cs-137 source and the vertical distance between the source and fungus.

A review of previous studies was conducted to identify the gamma dose rate and total dose that should be targeted for exposure (Table S2). Those dose rates ranged from 600,000 rad delivered in 1.5 h to 0.08 rad delivered in 16 h. Even among studies examining the same fungi attributes, the total dose varied dramatically. For the present study, we used a Health Physics code to target a 50-rad dose over a one-week exposure. This dose was selected as it changes blood count observed in most humans^24^. We hypothesized that this dose would induce physiological changes in the fungi without causing a high rate of lethality. A MicroShield (Grove Software, Inc.) model was created to identify the quantity of radioactive material and distance between source and sample necessary to achieve the dose of 50 rad in seven days. From a sensitivity analysis of the MicroShield model, it was determined that ~ 350 µCi of Cs-137 would create a dose rate of ~ 50 rad in seven days (Fig. 1; Table S3), if placed 1.8 cm from the surface of the fungi. It should be noted that Microshield values are often conservative and likely underestimate the actual dose on target. In addition, 50 rad falls in the middle of the large range for energies previously reported in the general literature (Table S2).

**Figure 2:**
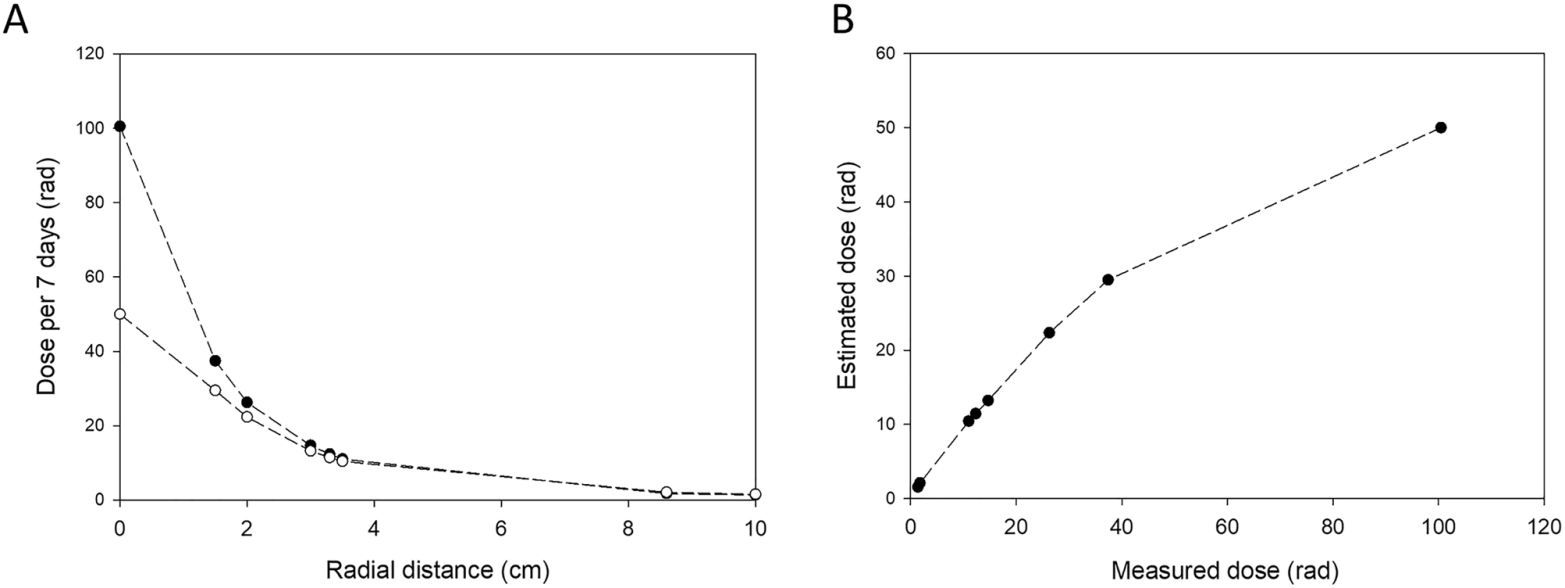
(**A**) The total gamma dose on the fungal mycelial at 7 days as a function of the radial distance from the central mycelium plug based on empirical measurements (-●-) and estimated from simulations (-○-). (**B**) Observed correlation between the measured and estimated doses at varying radial distances.

The dose from the Cs-137 source on the fungal mycelium is also dependent on the radial growth of the fungus from the center plug used to initiate growth. As the fungus grows away from the source, the leading edge will experience a lower total dose of radiation. Although a uniform dose would have been ideal, a source with activity sufficient to create a uniform radiation field would have initiated a variety of safety controls deemed impractical for this experiment. The background radiation dose at the testing site in Albuquerque is approximately 10 µrem h^−1^; the dose at the outermost area of the Petri dish was measured at 65,553 µrad h^−1^. As this dose was primarily from gamma emissions, rad and rem can be considered equivalent. To validate the simulation, a dose rate study was performed using thermoluminescent dosimeters (TLD) placed at varying distances from the center of the source. The TLD placed directly under the source measured ~ 100 rad over the seven-day exposure, which is double the prediction from the simulation (50 rad; Fig. 2A). However, at a radial distance of 3.5 cm, the measured and estimated total dose over seven days were much closer, 12.3 and 11.4 rad, respectively. A comparison of the measured and estimated dose on target demonstrated a non-linear correlation (Fig. 2B), in which the simulation better approximated the dose at larger radial distances from the source.

**Figure 3:**
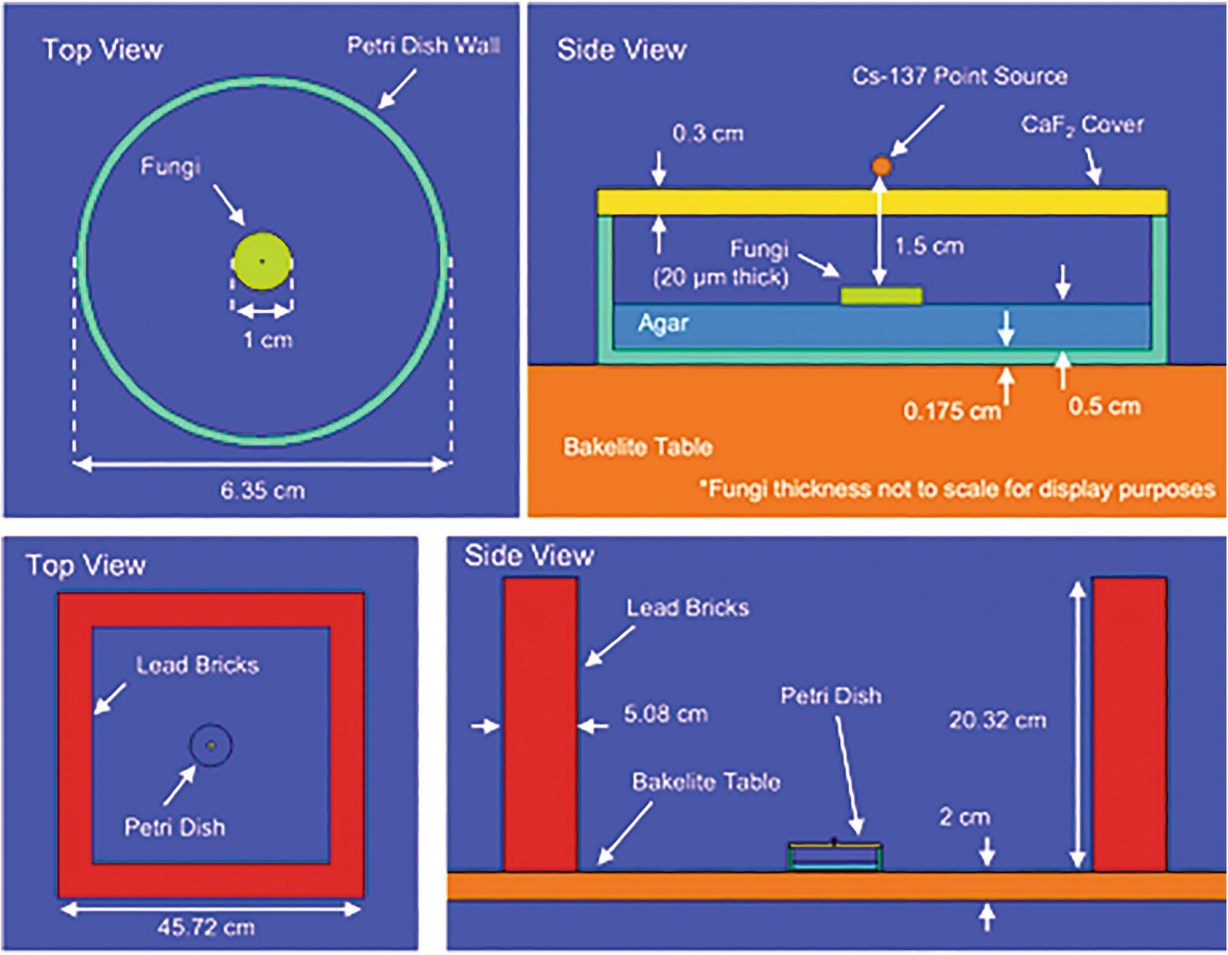
Top (upper left) and side (upper right) view of the Petri dish and fungi materials and distances used to determine energy deposition rates in MCNP. The overall geometry used for the radiation transport simulations, including the lead bricks, is shown from the top down (lower left) and from the side (lower right).

In order to normalize the energy deposited in the fungi from Cs-137 and the UV lamp sources, the units of MeV g^−1^ s^−1^ were selected for additional simulations. Monte Carlo N-Particle transport code (MCNP) simulations were used to determine this quantity for the Cs-137. The materials and geometry of the Petri dish and fungus used for these simulations are shown in Fig. 3. The Cs-137 was simulated as a point source located 1.5 cm from the top of the fungi. The Petri dish was set on a bakelite table. The setup was located in the center of a notional 5 m × 5 m × 5 m room with 30 cm thick concrete walls and filled with air. Leads bricks set on the table surrounded the petri dish and source. The International Commission on Radiological Protection (ICRP) material definitions did not contain data for fungal mycelia. Thus, we selected for skin as the closest approximation of the properties of the fungal mycelium^25^. This simulation gave a result for the energy deposited per particle as 6.53 × 10^–4^ MeV g^−1^, which for a 350 μCi activity, the rate of energy deposition was determined to be 7907 MeV g^−1^ s^−1^.

### UV source and irradiation

Our intent was to match the energy absorbed by the fungi to control for all variables except the photon energy difference between the Cs-137 source and UV lamp. The spectrum of energies emitted from the Cs-137 source varied significantly from those of the UV lamp, which in this case was a 30 W deuterium lamp that emitted from 185 to 400 nm (Fig. S1). This wide bandwidth represented photon energies ranging from 3.1 to 6.7 eV. The bandwidth of the UV exposure was limited to 300–350 nm using a 50-nm bandpass filter centered at 325 nm to ensure that incident photons would be in the UV energy range and not form ozone. Because we chose to match the overall energy deposited from the UV source to the gamma source it was necessary to attenuate the beam to the right power level. We assumed that all the UV energy would be absorbed near the surface rather than in the bulk since the fungi were melanized. This simplified the calculations and reduced risk, given the challenge of accurately estimating the absorbance of the fungi. The power deposited by the gamma source was calculated as the rate of energy deposition was determined to be 7907 MeV g^−1^ s^−1^ (1.3 nW g^−1^ s^−1^). Given the initial size of the plug was 1 cm in diameter, the desired lamp fluence needed to be ~ 2.8 nW cm^−2^. Across the spectrum of interest, the lamp power was determined to be 3.202 × 10^–4^ mW, thus requiring an attenuation of 8.7 × 10^–9^ (OD 8.06), reducing the lamp power to ~ 3 pW cm^−2^ and achieving a reasonably close power density to the target. Due to the sensitivity of UV detectors, the required power densities could not be measured directly. Alternatively, we measured the neutral density filters to verify the prescription was indeed correct.

### Response of *P. variotii *to irradiation

Uniform plugs (~ 5-mm in diameter) of actively growing mycelia of *P. variotii* were cut using the end of a Pasteur pipette and transferred a Petri plate containing potato dextrose agar (PDA) one day prior to initiating exposure experiments. The diameter of the mycelium was measured from four images, separated by precisely six hours, over the course of seven days and used to measure the growth rate. Differences in the pigmentation of the fungi under the different conditions was quantified in Fiji^26^ through analysis of grayscale images collected at day seven, following the method described by Brilhante et al.^27^ A ratiometric value was derived from the grayscale values and the white background, which corrected for variations in lighting across or between images.

**Figure 4:**
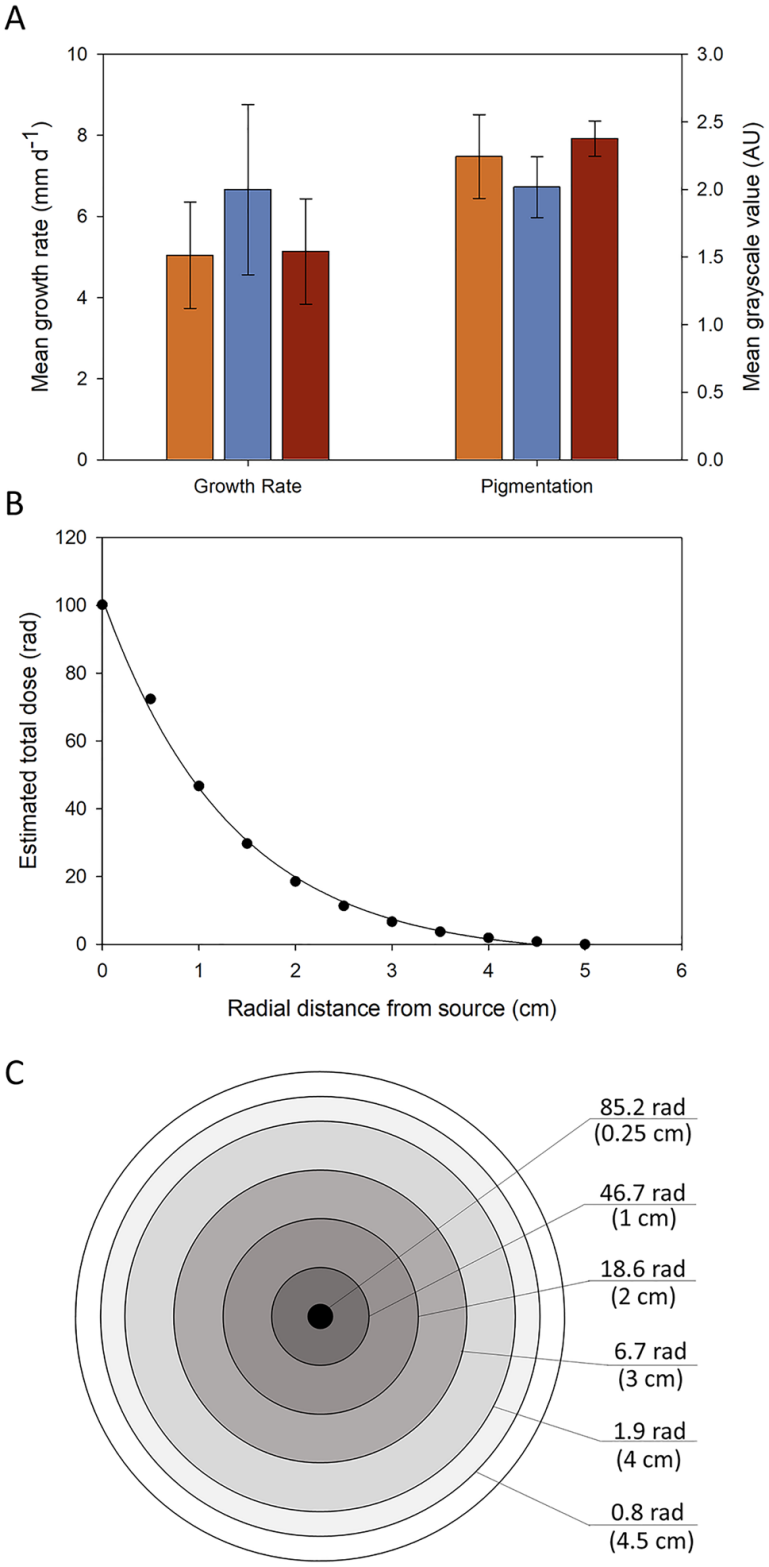
(**A**) Growth rate and pigmentation of control (orange square), gamma- (blue square), and UV- (red square) irradiated cultures of *P. variotti* (mean ± standard deviation)*.* (**B**) Estimated total irradiation dose experienced by the mycelial as a function of the distance from the central source. Exponential decay fit: − 3.6 + 105.7*exp(− 0.75*x); Adjusted R^2^ = 0.998. (**C**) Graphical representation of the irradiation dose based on the growth rate and duration of exposure for zones of mycelia as a function of radial distance from the central plug.

Significant differences in the pigmentation but not growth rates of *P. variotii* were associated with exposure to UV and gamma to irradiation, based on One-Way ANOVA analyses (Fig. 4A; Table S4). *P. variotii* is a ubiquitous filamentous fungus commonly inhabiting soil, decaying plants, and food products and was reported to be present on the surface of the walls of Unit-4 at ChNPP^22,28^. *P. variotii* is also a common food contaminant and is resistant to high temperature and metals^29,30^, despite being more sensitive to gamma irradiation than other fungi such as *Aspergillus fumigatus*^31^. In the present study, we hypothesized that positive radiation-induced effects in *P. variotii* would result in enhanced growth rates due to gamma irradiation. Across all conditions, the average growth rate of *P. variotii* was ~ 5.6 ± 0.9 mm d^−1^ (mean ± standard deviation). While the growth rate of *P. variotii* exposed to gamma irradiation was greater compared with the control and UV-irradiated samples (Fig. 4A), the difference in the mean growth rates was not significant (P = 0.255) by ANOVA.

We also hypothesized that the pigmentation of *P.* *variotii* would increase with exposure to gamma and UV irradiation. While *P. variotti* does not produce melanin, it does produce a pigment, Ywa1, from a polyketide synthesis (PKS) gene cluster and has been shown to protect the fungus against UV-C irradiation^28^. In some melanized fungi, Ywa1 serves as precursor and can be hydrolyzed to 1,3,6,8-tetrahydroxynaphthalen (T4HN). T4HN may then be converted to 1,8-dihydroxynaphthalene (1,8-DHN) melanin through the DHN pathway^32^. However, Lim et al.^28^ concluded that *P. variotii* does not produce true melanin as the pigmentation was maintained when the DHN-melanin pathway was inhibited. Significant differences in the pigmentation of *P. variotii* were observed among the three different sample types (P < 0.001; Table S4). Here, the gamma irradiation produced the least amount of pigmentation, followed by the control, and the UV-irradiated produced the greatest level of pigmentation (Fig. 4A). The high level of pigmentation in the UV-irradiated samples is consistent the prior report of enhanced PKS production to protect against UV-C exposure^28^. Further, the lower levels of pigmentation in gamma irradiated samples of *Paecilomyces* are consistent with its reported sensitivity to gamma irradiation^31^.

Because the fungus grew radially outward from a plug placed at the center of the Petri plate, the total dose on a given region of the fungus is dependent upon both the distance from the source and the duration that a specific region is exposed. For example, *P. variotii* grew at an average rate of 6.7 mm d^−1^, almost completely covering the surface of a Petri plate. Using the measured growth rate value and the dose at given distances away from the source, based on TLD data, we estimated the total dose of the fungal mycelium as it grew from the central plug (Fig. 4B), which displayed an exponential decrease as a function of distance from the plug. Figure 4C provides a graphical representation of this effect, where each 1-cm ring of fungal mycelium experienced considerably less gamma dose than the prior ring. This relationship is critical to evaluate within the context of radiotropism/radiotolerance, as the total dose on the mycelium is anisotropic. As such, in our experiment, the leading growth edge of the fungus is continuously moving away from the irradiation source and experiencing smaller and smaller total doses of radiation.

### Response of *C. cladosporioides* to irradiation

**Figure 5:**
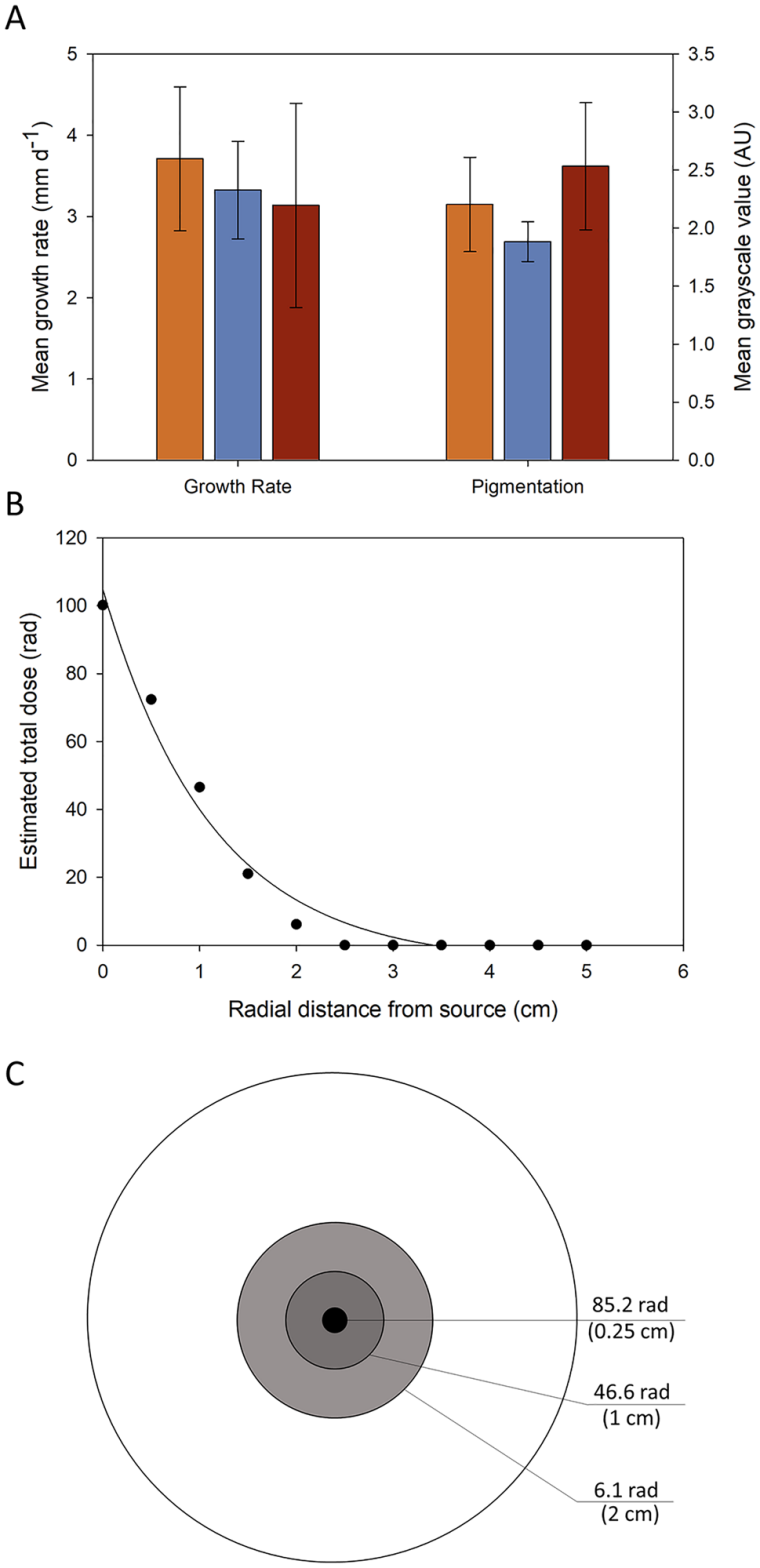
(**A**) Growth rate and pigmentation of control (orange square), gamma- (blue square), and UV- (red square) irradiated cultures of *C. cladosporioides* (mean ± standard deviation)*.* (**B**) Estimated total irradiation dose experienced by the mycelial as a function of the distance from the central source. Exponential decay fit: − 5.3 + 110.1*exp(− 0.89*x); Adjusted R^2^ = 0.974. (**C**) Graphical representation of the irradiation dose based on the growth rate and duration of exposure for zones of mycelia as a function of radial distance from the central plug.

Similar to the results for *P. variotii,* significant differences in the pigmentation, but not the growth rate of *C. cladosporioides* were observed in samples exposed to gamma or UV irradiation (Fig. 5; Table S5). *C. cladosporioides* is a melanized filamentous fungus that is pervasive in outdoor and indoor air, and on decaying organic matter^33^. Isolates of *C. cladosporioides* were collected from the walls of Unit-4 of the Chernobyl power plant in locations with both weak and severe levels of radioactive contamination^22^. Reports further suggest that *C. cladosporioides* exhibits radiotropism, i.e., preferential growth toward radioactive particles^3,5,34,35^. This behavior has been linked to the high production of melanin pigments by *Cladosporium* species, which not only protect the fungus from ionizing radiation, but also may enable its exploitation as an energy source^13,17^. A radiotrophic isolate of *C. cladosporioides* from ChNPP, IMV 00236, was also reported to have increased production of several secondary metabolites following irradiation with 200–300 nm light for 30 min, suggesting physiological changes potentially related to radiotropism^36^. With respect to the latter, melanized cells of *C. sphaerospermum* displayed enhanced growth under limited nutrients and with radiation from a ^188^Re/^188^W generator^17^. In the present work, the average growth rate of *C. cladosporioides* across all treatments was 3.4 ± 0.3 mm d^−1^, and no significant differences in the growth rate were observed due to irradiation (P = 0.615; Table S5).

Significant differences in the pigmentation of *C. cladosporioides* cultures were observed among the different treatments (P < 0.001; Table S5). The darkest pigmentation was observed for cultures exposed to UV irradiation, followed by the control, and then the gamma-irradiated sample (Fig. 5A). The observation for high pigmentation in UV-irradiated samples is consistent with the role of melanin in protecting fungi from UV^37^. *Cladosporium* produces melanin by through the DHN pathway and has been implicated in radiotolerance/radiotropism^17,38,39^. The lower level of pigmentation in gamma irradiated samples, however, contrasts prior reports that suggest melanin provides enhanced protection against gamma irradiation^13,37,40^. Similarly, gamma irradiation has been reported to change the electronic properties of melanin^41^, allowing it to be used as an energy source in melanized fungi^13,17^. In our work, *C. cladosporioides* produced significantly lower levels of pigments when exposed to gamma irradiation as compared to the control and UV-irradiated samples (P < 0.01; Table S5). This difference, at least in part, may be related to the fact that this environmental isolate had not been directly exposed to a gamma-rich environment prior to this experiment, compared to those isolated from ChNPP, which were exposed to radiation for years to decades. In addition, fungi in all of our experiments were grown on nutrient rich media (PDA). In prior work, Dadachova et al.^17^ reported enhanced growth *C. sphaerospermum* and *Wangiella dermatitidis* induced by ionizing radiation under conditions of limited nutrient availability. Thus, the nutrient-rich conditions in our experiments may have masked potential radiation-induced enhancement in growth.

As noted above, the radiation exposure profile may be estimated based on the growth rate of the fungus and the total dose on target, which is dependent on the distance from the source and duration on target. The average growth rate of *C. cladosporioides* exposed to gamma irradiation in our experiments was 3.3 mm d^−1^ (Fig. 5A), which was approximately half the rate observed for *P. variotii* (6.7 mm d^−1^; Fig. 4A). As such, the radiation exposure profile for *C. cladosporioides* (Fig. 5B,C) is also considerably different. Here the total dose on target ranged from 85.2 rad at the central mycelial plug to 6.1 rad as the growing edge of the mycelium (Fig. 5C), approximately 2 cm from the central plug. In contrast, the growing edge of *P. variotii* experienced a much lower total dose, < 1 rad at 4.5 cm from the central plug. This difference is important to consider when comparing the response of fungi to radiation as the growing edge of the mycelium is metabolically active while the older hyphae become vacuolated and lose their cytoplasm.

## Conclusions

We studied the response of *C. cladosporioides* (melanized) and *P. variotii* (non-melanized) to equivalent total dose of gamma and UV irradiation to understand the role of different photon energies on the phenomena associated with radioprotection, including radiotolerance, radiotropism, and radiosynthesis. Significant design and modeling efforts were invested in the control of normalizing photon absorption across photon energies, focused on mitigating the confounding variables that could affect the experimental outcome. While differences in growth rate were not observed, differences in the pigmentation of both fungi were observed following both UV and gamma exposure. Most notably, decreased pigmentation in *C. cladosporioides* and *P. variotii* was observed in response to gamma irradiation. This result was unexpected as melanin production in fungi such as *C. cladosporioides* has been hypothesized to protect and potentially enhance the growth of melanized fungi in the presence of gamma irradiation. Past studies reporting differential growth and melanin production for gamma-irradiated fungi as compared to a control indicate widely varied exposure intensity and duration. It is possible that radiation-induced changes are dependent on select photon energy, higher total dose, and/or evolutionary adaptation of specific fungal strains. These factors were beyond the scope of the present study but should be considered in follow-on work. Future studies also could include the use of radionuclides emitting a wider range of energies or a different type of ionizing radiation, such as neutron, alpha, or beta. Finally, examining growth effects using fungi samples whose evolution is directed toward a radiotrophic behavior through sequential culture and passage of mycelium under continuous irradiation could provide additional understanding to radionuclide-fungal interactions.

## Materials and methods

### Radiation modeling

From a review of the of the literature, it was determined that, to provide strong evidence of radiotropism, efforts must focus on ensuring control of environmental variables and duration and intensity of the exposure and normalization of absorption across radiation types. To meet these objectives, the experimental design process used modeling and simulation tools to understand the radiation environments to which the fungi would be exposed, and to ensure that fungi photon absorption was held constant across energies.

MicroShield (Grove Software, Inc.) was used to create a model to quantify the radioactive material and distance between source and sample necessary to achieve the dose of 50 rad in seven days. Using this model, we determined the distance of the source above the fungal plug necessary to achieve the 50-rad total dose. The model was then applied to determine the dose on target as a function of the radial distance away from the source. The simulations were validated with dose rate study using thermoluminesent dosimeters (TLDs) placed at varying distances from the center of the source.

A key goal of our work was to normalize the energy deposited on the fungi from Cs-137 and the UV lamp sources. Monte Carlo N-Particle transport code (MCNP) simulations were used to determine this quantify the dose rate for the Cs-137. Once this value was determined, a UV excitation source (i.e., 30 W deuterium lamp that emitted from 185 to 400 nm) and a 50 nm bandpass filter centered at 325 nm were selected, and the energy deposited on the fungal target was estimated. These calculations indicated that a filter of OD ~ 8.06) was necessary to attenuate the beam to achieve an equivalent total dose over seven days.

### Experimental setup

To best mitigate potential differences due to environmental variables, all samples and exposures were colocalized in a single test system (Fig. S6, S7). Gamma photons were shielded with lead bricks to prevent irradiation of control and UV samples. The efficacy of this shielding was assessed with the use of Grove MicroShield modeling and validated by physical measurement once the source was in place. UV photons directed to the sample as shown in Fig. S8, and controlled by the use of acrylic boxes. Two LED light strips of the same manufacturer and output specifications were used to illuminate each sample for the purpose of photography. Humidity and temperature were logged, and images were collected of the gamma, UV, and control samples every ten minutes, for the duration of the experiment, and used to calculate growth rates as described below. To mitigate the risk of confounding variables during this experiment, significant upfront energy was invested in the experimental design and operational setup.

### Fungal cultures and test samples

**Figure 6:**
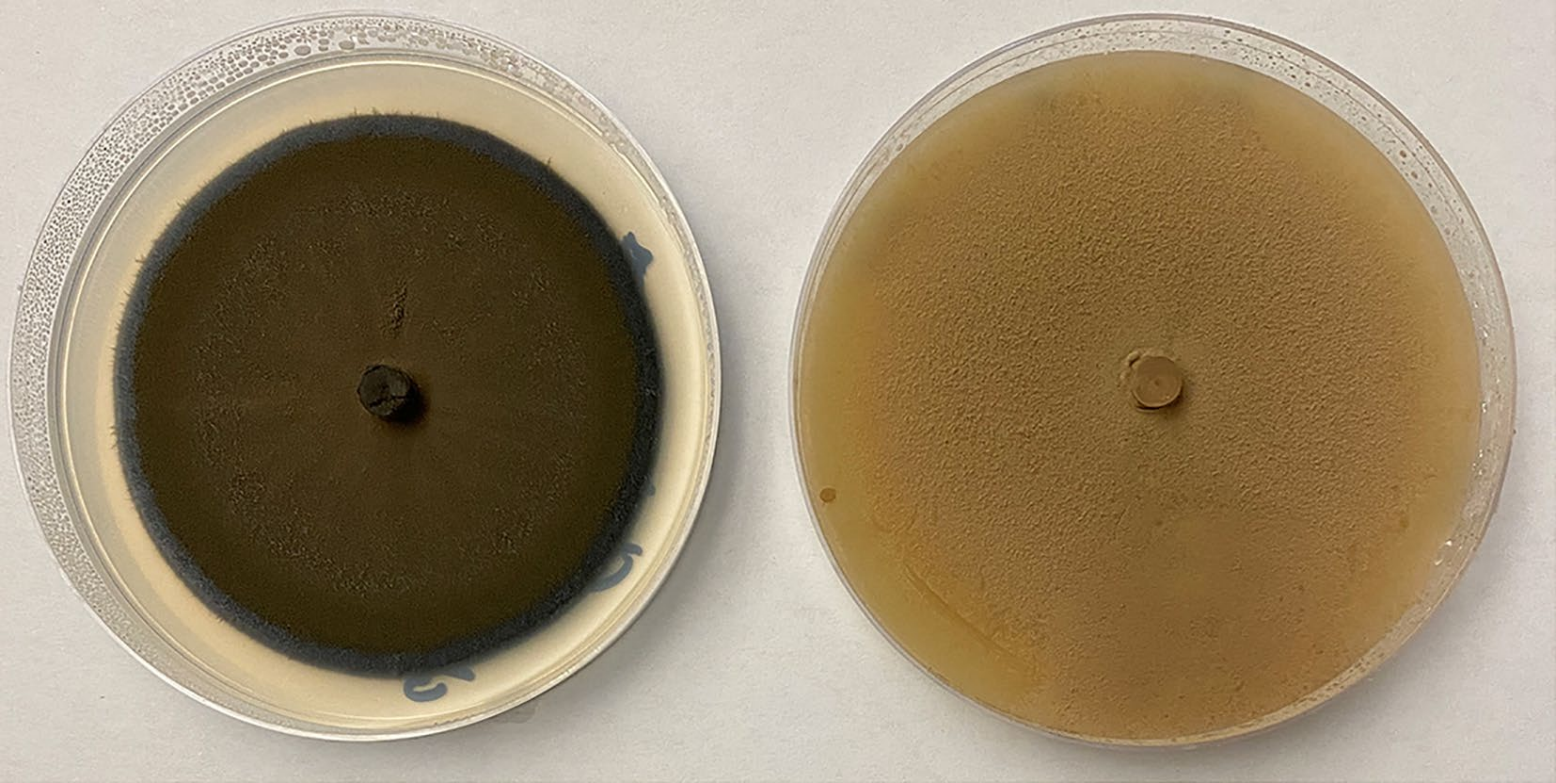
Cultures of (left) *C. cladosporioides* and (right) *P. variotti*.

Stock cultures of *P. variotii* or *C. cladosporioides* (Fig. 6) were derived from environmental isolates from facilities at Sandia National Laboratories in Albuquerque, NM. DNA sequencing was used for identification and confirmed by microscopic analysis. Starting cultures of both fungi were cultured for seven days on potato dextrose agar (PDA) at 30 °C. Uniform plugs (five-mm diameter) of mycelia were cut using the end of a Pasteur pipette. Plugs were then transferred to a new PDA plate one day prior to initiating exposure experiments. The Petri plate covers were replaced with a quartz disc (101.6 mm × 3.2 mm, #16004-2, Ted Pella, Inc.) to minimize attenuation of the UV photons, and sealed with Parafilm. On Day 0, Petri plates were moved into the test system and exposure to the gamma or UV source was initiated. Photographs of the plates were obtained every ten minutes over seven days. The diameter of the growing mycelial mat was measured on four images, separated by precisely six hours per day over the course of the seven-day exposure for each experimental treatment. The growth rate was determined using linear regression analysis.

Additional photographs were obtained on day seven to characterize differences in pigmentation due to the different treatments. Changes in the pigmentation of the two fungi under the different conditions was quantified by analysis of grayscale images, based on the method described by Brilhante et al.^27^ Briefly, photographs were first converted into grayscale using Fiji^26^ and mean gray value (0–250) was collected on five regions of interest (ROIs) on each fungal sample and treatment. The mean gray values (n = 5 ROIs) of the white background also were collected to correct for any variation in lighting across the image. A ratiometric value was then calculated by dividing the mean gray values of each fungal sample by the mean gray value of the background.

## Supplementary Information

Supplementary Information.

## Data Availability

The datasets used and/or analyzed during the current study available from the corresponding author on reasonable request.
