# Supplementary material for: Evaluating changes in growth and pigmentation of Cladosporium cladosporioides and Paecilomyces variotii in response to gamma and ultraviolet irradiation
Source: Sci Rep. 2022 Jul 15;12:12142. doi: 10.1038/s41598-022-16063-z (PMC9287308; doi:10.1038/s41598-022-16063-z)
Supplement: Supplementary file 1 — Supplementary Information. [file 41598_2022_16063_MOESM1_ESM.pdf]

Evaluating changes in growth and pigmentation of *Cladosporium cladosporioides* and *Paecilomyces variotii* in response to gamma and ultraviolet irradiation

Jesse Bland,[1] Lisa Astuto Gribble,[1] Michael C. Hamel,[1] Jeremy B. Wright,[2] Garrett Moormann,[3] Marlene Bachand,[3] Ginger Wright,[1] and George D. Bachand[3]*

[1]Center for Global Security and Cooperation, [2]Center for Monitoring Systems & Technology, and [3]Center for Integrated Nanotechnologies, Sandia National Laboratories, Albuquerque, NM 87185 USA

*E-mail: gdbacha@sandia.gov

Table S1. Yield per decay for the different photon energies emitted by Cs-137 source.

Energy (keV)	Yield per Decay (%)
661.657	85.325
32.194	3.979
31.817	2.16
36.378	0.7198
36.304	0.3729
37.255	0.2279
37.349	5.09×10^{-2}
36.652	8.4×10^{-3}
283.5	5.8×10^{-4}
31.452	2.79×10^{-4}

Table S2. Summary of reported responses of fungi to ionizing radiation.

Particle	Source	Energy	Dose Rate (rad/h)	Time of Exposure (h)	Total Dose (rad)	Quantity Measured	Citation
***Cryptococcus neoformans* (melanized and non-melanized)**							
Photon	X-ray	150 kVp	12.1	24	290	CFU/ml	1
Photon	X-ray	150 kVp	251	24	6024	CFU/ml	1
Photon	X-ray	150 kVp	12.1	48	581	CFU/ml	1
Photon	X-ray	150 kVp	251	48	12048	CFU/ml	1
Photon	X-ray	320 kVp	38.3	24	919	CFU/ml	1
Photon	X-ray	320 kVp	549	24	13176	CFU/ml	1
Photon	X-ray	320 kVp	38.3	48	1838	CFU/ml	1
Photon	X-ray	320 kVp	545	48	26160	CFU/ml	1
Photon	X-ray	320 kVp	0.012	48	0.58	CFU/ml	1
Photon	Re-188/W-188 isotope generator	155 keV (and other higher energy low probability gammas)	0.005	overnight	unknown	Number of electron-transfer events	2
Photon	Re-188/W-188 isotope generator	155 keV (and other higher energy low probability gammas)	0.005	18	0.09	CFU/ml	2
Photon	Re-188/W-188 isotope generator	155 keV (and other higher energy low probability gammas)	0.005	23	0.12	CFU/ml	2
Photon	Re-188/W-188 isotope generator	155 keV (and other higher energy low probability gammas)	0.005	30	0.15	CFU/ml	2
Photon	Re-188/W-188 isotope generator	155 keV (and other higher energy low probability gammas)	0.005	18	0.09	Uptake of ^{14}C-acetate	2
Photon	Re-188/W-188 isotope generator	155 keV (and other higher energy low probability gammas)	0.005	23	0.12	Uptake of ^{14}C-acetate	2

Particle	Source	Energy	Dose Rate (rad/h)	Time of Exposure (h)	Total Dose (rad)	Quantity Measured	Citation
Photon	Re-188/W-188 isotope generator	155 keV (and other higher energy low probability gammas)	0.005	30	0.15	Uptake of ^{14}C-acetate	[2]
***C. neoformans* (melanized)**							
Photon	Co-60	1173 and 1332.5 keV	30000	1	30000	Change in electrical current (I/nA)	[3]
Photon	Co-60	1173 and 1332.5 keV	400000	1	400000	Change in electrical current (I/nA)	[3]
Photon	Co-60	1173 and 1332.5 keV	400000	1.5	600000	Change in electrical current (I/nA)	[3]
Photon	Co-60	1173 and 1332.5 keV	60000	0.28	16800	Change in melanin oxidation-reduction potential (E/V)	[3]
Photon	Cs-137	662 keV	0.233	0.33	0.078	Rate of NADH/ferricyanide reaction	[2]
Photon	Cs-137	662 keV	0.233	0.67	0.155	Rate of NADH/ferricyanide reaction	[2]
Photon	UV	4.7 eV	N/A	0.67	N/A	Rate of NADH/ferricyanide reaction	[2]
Photon	Visible Light	3 eV	N/A	0.67	N/A	Rate of NADH/ferricyanide reaction	[2]
Photon	Heat	0.1 eV	N/A	0.67	N/A	Rate of NADH/ferricyanide reaction	[2]
***Cladosporium sphaerospermum* (melanized)**							
Photon	Re-188/W-188 isotope generator	155 keV (and other higher energy low probability gammas)	0.005	192	0.96	Volume (mm^3)	[2]

Particle	Source	Energy	Dose Rate (rad/h)	Time of Exposure (h)	Total Dose (rad)	Quantity Measured	Citation
Photon	Re-188/W-188 isotope generator	155 keV (and other higher energy low probability gammas)	0.005	288	1.44	Volume (mm^3)	2
Photon	Re-188/W-188 isotope generator	155 keV (and other higher energy low probability gammas)	0.005	360	1.8	Volume (mm^3)	2
Photon	Re-188/W-188 isotope generator	155 keV (and other higher energy low probability gammas)	0.005	90	0.45	Radial growth rate (mm/hr)	2
Photon	Re-188/W-188 isotope generator	155 keV (and other higher energy low probability gammas)	0.005	190	0.95	Radial growth rate (mm/hr)	2
Photon	Re-188/W-188 isotope generator	155 keV (and other higher energy low probability gammas)	0.005	290	1.45	Radial growth rate (mm/hr)	2
***Exophiala* (*Wangiella*) *dermatitidis* (wild melanized, melanized, and non-melanized)**							
Photon	Re-188/W-188 isotope generator	155 keV (and other higher energy low probability gammas)	0.005	8	0.04	CFU/ml	2
Photon	Re-188/W-188 isotope generator	155 keV (and other higher energy low probability gammas)	0.005	16	0.08	CFU/ml	2
Photon	Re-188/W-188 isotope generator	155 keV (and other higher energy low probability gammas)	0.005	22	0.11	CFU/ml	2
Photon	Re-188/W-188 isotope generator	155 keV (and other higher energy low probability gammas)	0.005	30	0.15	CFU/ml	2
Photon	Cs-137	662 keV	0.01 (beam), 5e-5 (cells)	unknown	0.000015 - 0.000025 (cells)	Relative colony size	4

Particle	Source	Energy	Dose Rate (rad/h)	Time of Exposure (h)	Total Dose (rad)	Quantity Measured	Citation
Alpha	Po-210	5304 keV	unknown	unknown	unknown	Relative colony size	4
Beta	Sr-90	546 keV (end-point energy)	6E-5 (cells)		0.00002 - 0.00003 (cells)	Relative colony size	4

Table S3. Time required on target to achieve 50 rad based on an activity of ~350 µCi for Cs-137 source and the vertical distance between the source and fungus.

Distance (cm)	Dose rate (rad h^{-1})	Time on target for 50 rad (d)
0.798	0.70	1.4
1.798	0.30	6.9
2.798	0.12	16.8
3.798	0.07	30.9
4.798	0.04	49.3
5.798	0.03	71.9
6.798	0.02	98.9
7.798	0.02	130.1
8.798	0.01	165.7
9.798	0.01	205.5
10.798	0.01	249.6

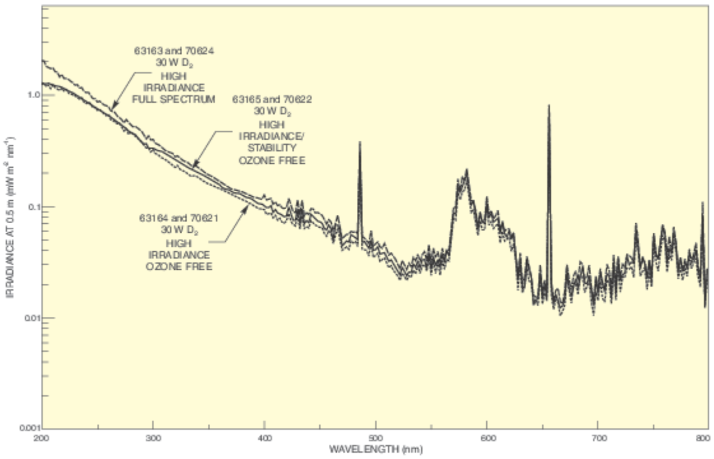

Figure S1. Full spectral irradiance of the UV lamp (MODEL 63165) as provided by Newport (https://www.newport.com/p/63165).

Table S4. Results from One-Way ANOVA analyses of the growth rate and grayscale value of control, gamma- and UV-irradiate samples of *Paecilomyces variotti.*

Growth Rate One-Way ANOVA

Group Name	N	Mean	Std Dev	SEM
Control	6	5.045	1.311	0.535
Gamma	4	6.662	2.097	1.049
UV	5	5.138	1.298	0.581

Source of Variation	DF	SS	MS	F	P
Between Groups	2	7.290	3.645	1.533	0.255
Residual	12	28.527	2.377		
Total	14	35.817			

Grayscale Value One-Way ANOVA

Group Name	N	Mean	Std Dev	SEM
Control	50	2.243	0.310	0.0439
Gamma	25	2.017	0.225	0.0450
UV	25	2.376	0.131	0.0262

Source of Variation	DF	SS	MS	F	P
Between Groups	2	1.664	0.832	12.725	<0.001
Residual	97	6.343	0.0654		
Total	99	8.008			

All Pairwise Multiple Comparison Procedures (Holm-Sidak method):
Overall significance level = 0.05

Comparison	Diff of Means	t	P	P<0.050
Control vs. Gamma	0.226	3.611	<0.001	Yes
Control vs. UV	0.133	2.118	0.037	Yes
UV vs. Gamma	0.359	4.961	<0.001	Yes

Table S5. Results from One-Way ANOVA analyses of the growth rate and grayscale value of control, gamma- and UV-irradiate samples of *Cladosporium cladosporioides.*

Growth Rate One-Way ANOVA

Group Name	N	Mean	Std Dev	SEM
Control	5	3.711	0.884	0.395
Gamma	6	3.325	0.600	0.245
UV	6	3.136	1.258	0.514

Source of Variation	DF	SS	MS	F	P
Between Groups	2	0.923	0.462	0.503	0.615
Residual	14	12.841	0.917		
Total	16	13.765			

Coloration One-Way ANOVA

Group Name	N	Mean	Std Dev	SEM
Control	50	2.204	0.406	0.0575
Gamma	25	1.883	0.171	0.0342
UV	25	2.532	0.548	0.110

Source of Variation	DF	SS	MS	F	P
Between Groups	2	5.271	2.636	15.973	<0.001
Residual	97	16.005	0.165		
Total	99	21.276	99		

All Pairwise Multiple Comparison Procedures (Holm-Sidak method):
Overall significance level = 0.05

Comparison	Diff of Means	t	P	P<0.050
Control vs. Gamma	0.321	3.226	0.002	Yes
Control vs. UV	0.328	3.300	0.003	Yes
UV vs. Gamma	0.649	5.652	<0.001	Yes

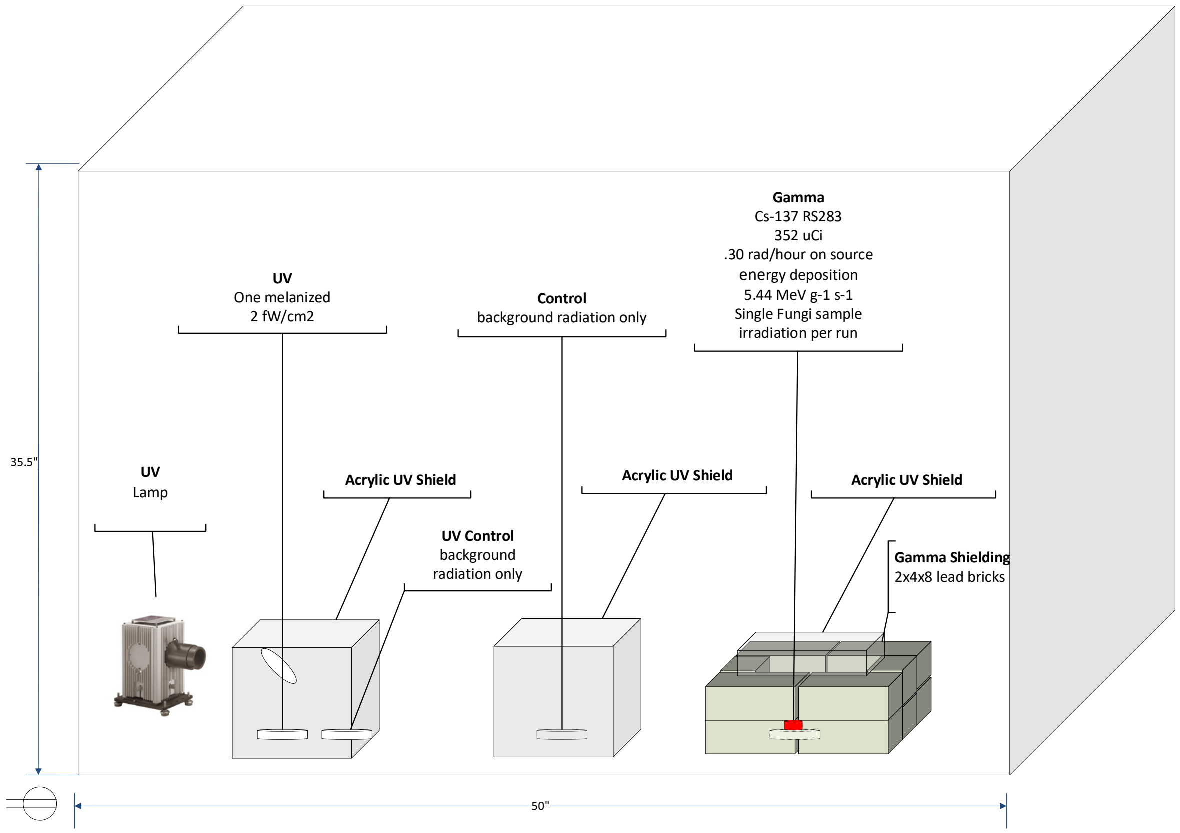

Figure S6. Sketch of experimental system.

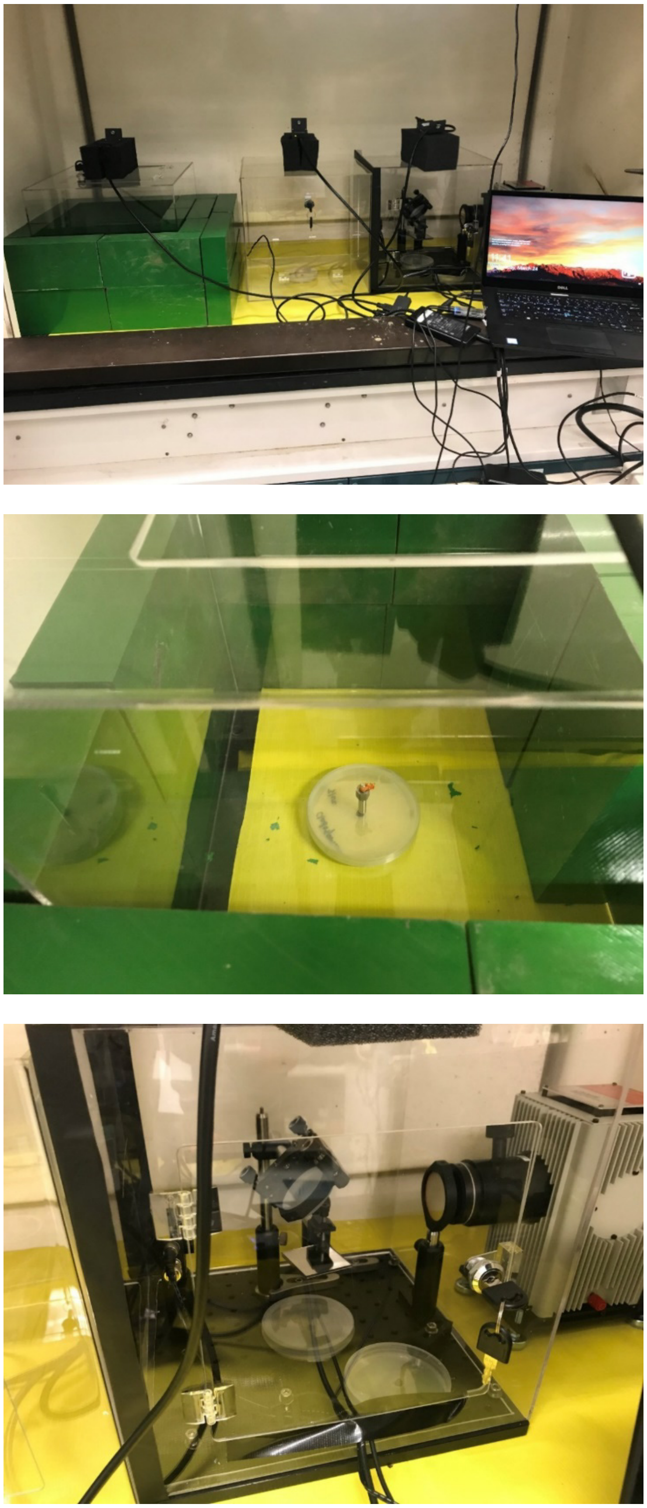

Figure S7. (top) Overview of experimental system. (middle) Cs-137 gamma source on Petri plate containing fungal plug. (bottom) UV source irradiating fungal sample. Control sample (non-irradiated) is visible adjacent to the irradiated sample.

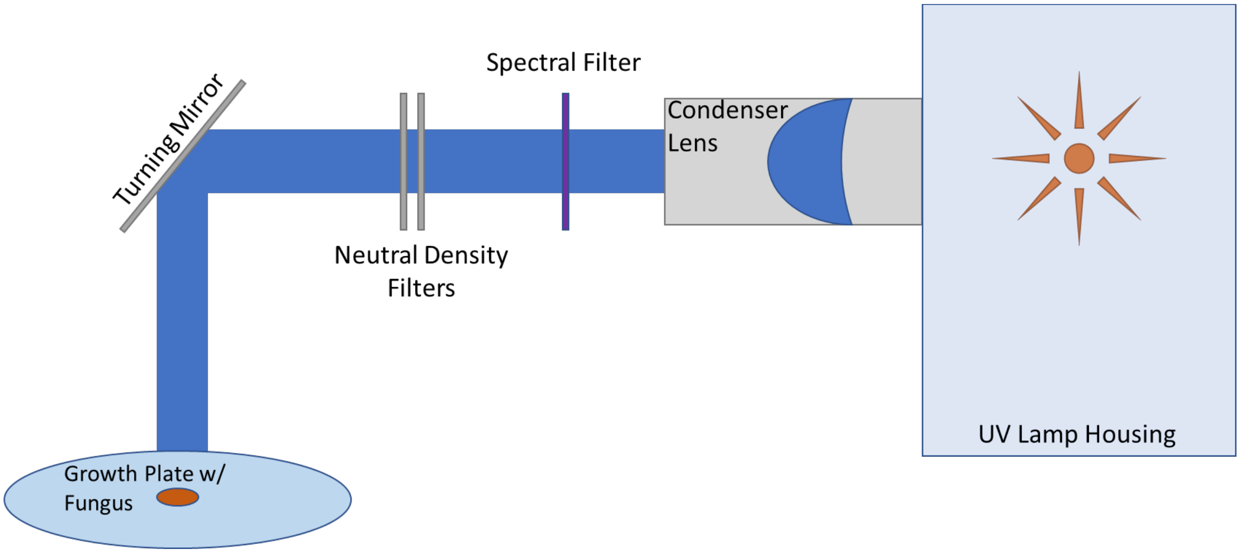

Figure S8. Schematic diagram of the UV setup showing the UV lamp and optical components.

References

1. Shuryak, I.; Bryan, R. A.; Nosanchuk, J. D.; Dadachova, E., Mathematical modeling predicts enhanced growth of X-ray irradiated pigmented fungi. *PLoS One* **2014,** 9, (1), e85561.
2. Dadachova, E.; Bryan, R. A.; Huang, X.; Moadel, T.; Schweitzer, A. D.; Aisen, P.; Nosanchuk, J. D.; Casadevall, A., Ionizing radiation changes the electronic properties of melanin and enhances the growth of melanized fungi. *PloS One* **2007,** 2, (5), e457-e457.
3. Turick, C. E.; Ekechukwu, A. A.; Milliken, C. E.; Casadevall, A.; Dadachova, E., Gamma radiation interacts with melanin to alter its oxidation–reduction potential and results in electric current production. *Bioelectrochemistry* **2011,** 82, (1), 69-73.
4. Malo, M. E.; Bryan, R. A.; Shuryak, I.; Dadachova, E., Morphological changes in melanized and non-melanized *Cryptococcus neoformans* cells post exposure to sparsely and densely ionizing radiation demonstrate protective effect of melanin. *Fungal Biology* **2018,** 122, (6), 449-456.
